# Evaluation of the biocompatibility and stability of allogeneic tissue-engineered cartilage in humanized mice

**DOI:** 10.1371/journal.pone.0217183

**Published:** 2019-05-20

**Authors:** Emeline Perrier-Groult, Eléonore Pérès, Marielle Pasdeloup, Louis Gazzolo, Madeleine Duc Dodon, Frédéric Mallein-Gerin

**Affiliations:** 1 Laboratory of Tissue Biology and Therapeutic Engineering (LBTI), CNRS-UMR5305, Lyon, France; 2 Laboratory of Biology and Modeling of the Cell, Ecole Normale Supérieure (ENS) de Lyon, INSERM U1210, CNRS UMR5239, Lyon, France; Università degli Studi della Campania, ITALY

## Abstract

Articular cartilage (AC) has poor capacities of regeneration and lesions often lead to osteoarthritis. Current AC reconstruction implies autologous chondrocyte implantation which requires tissue sampling and grafting. An alternative approach would be to use scaffolds containing off-the-shelf allogeneic human articular chondrocytes (HACs). To investigate tolerance of allogeneic HACs by the human immune system, we developed a humanized mouse model implanted with allogeneic cartilage constructs generated *in vitro*. A prerequisite of the study was to identify a scaffold that would not provoke inflammatory reaction in host. Therefore, we first compared the response of hu-mice to two biomaterials used in regenerative medicine, collagen sponge and agarose hydrogel. Four weeks after implantation in hu-mice, acellular collagen sponges, but not acellular agarose hydrogels, showed positive staining for CD3 (T lymphocytes) and CD68 (macrophages), suggesting that collagen scaffold elicits weak inflammatory reaction. These data led us to deepen our evaluation of the biocompatibility of allogeneic tissue-engineered cartilage by using agarose as scaffold. Agarose hydrogels were combined with allogeneic HACs to reconstruct cartilage *in vitro*. Particular attention was paid to HLA-A2 compatibility between HACs to be grafted and immune human cells of hu-mice: HLA-A2^+^ or HLA-A2^-^ HACs agarose hydrogels were cultured in the presence of a chondrogenic cocktail and implanted in HLA-A2^+^ hu-mice. After four weeks implantation and regardless of the HLA-A2 phenotype, chondrocytes were well-differentiated and produced cartilage matrix in agarose. In addition, no sign of T-cell or macrophage infiltration was seen in the cartilaginous constructs and no significant increase in subpopulations of T lymphocytes and monocytes was detected in peripheral blood and spleen. We show for the first time that humanized mouse represents a useful model to investigate human immune responsiveness to tissue-engineered cartilage and our data together indicate that allogeneic cartilage constructs can be suitable for cartilage engineering.

## Introduction

An increased number of traumatic and degenerative lesions in articular cartilage (AC) has been observed with aging of the population. They have also raised in incidence in the younger population, due to intensive practice of sports. Since AC presents poor intrinsic healing potential, these lesions have high risk to evolve to osteoarthritis (OA), a worldwide leading cause of disability. Common surgical treatments (micro-fracture, mosaicplasty) often lead to the production of fibrocartilage which does not possess the biomechanical properties of AC. Besides, joint replacement is a short-term therapy because of the limited lifespan of knee prostheses.

AC is avascular and alymphatic and therefore could be considered as immunoprivileged that would allow implantation of allogeneic cells [1] and today, there is growing clinical demand for using allogeneic chondrocytes rather than autologous chondrocytes in the transplantation procedures to repair articular cartilage. However, several studies demonstrated that allogeneic transplantation of isolated chondrocytes triggers an immune response gradually destroying the regenerated cartilage [2,3]. These results stimulated the development of tissue-engineering approaches using allogeneic chondrocytes in combination with a wide variety of natural or synthetic scaffolds. The fate of transplanted allografts has been investigated for over three decades but still remains a matter of debate with conflicting results. To date, the *in vivo* repair capacity of allogeneic tissue-engineered cartilage has been evaluated only with animal models and mostly in rabbit. Rahfoth *et al*. [4] showed that implantation of rabbit allogeneic chondrocytes embedded in agarose hydrogel to form cartilage discs was not rejected and did not lead to immune cell infiltration. A study by Kawabe and Yoshinao has shown opposite results, with rejection of cartilage discs prepared with rabbit chondrocytes [5]. This intolerance was due to cell-mediated toxicity and humoral response accompanied with invasion of mononuclear cells throughout the graft and accumulation of lymphocytes around it. Mononuclear cells were also detected, although to a lesser extent, in grafts of allogeneic rabbit chondrocytes seeded in polyglycolic acid (PGA) meshes [6]. Of note, this cellular infiltration was maintained throughout the 24-month study with no sign of graft resorption or rejection. Although such pilot studies paved the way to analyze immune response to allogeneic tissue-engineered cartilage *in vivo*, they had limitations since species differences exist in immune cell receptors, cytokine expression or response to various stimuli. In the present study, we explored the humanized (hu) mouse model to investigate responsiveness to human allogeneic tissue-engineered cartilage. Hu-mice are generated by grafting human hematopoietic stem cells (HSC) isolated from cord blood into immunodeficient newborn mice. These hu-mice develop a human hemato-lymphoid system and offer a potent research model for investigating pathogenesis associated with infection by human lymphotropic viruses, for studying autoimmune diseases and reaction to xenogeneic transplantation and allogeneic stem cell transplantation [7–12]. Here, we used immunodeficient NSG-HLA-A2/HDD mice, created by backcrossing the HLA class I transgene (HDD construct designed for the expression of *A0201) onto the NSG background [13] to explore the stability and biocompatibility of human cartilage implants.

## Methods and materials

### Ethics statement

Anonymized human umbilical cord samples from the Maternity Ward of Hôpital Femme-Mère-Enfant (Bron, France) were obtained from healthy full-term newborns with written parental informed consent according to the guidelines of the medical and ethical committees of Hospices Civils de Lyon and of Agence de la Biomédecine (Paris, France). Experiments using cord blood samples were approved by both committees and were performed in full compliance with French law. Animal experimentation was performed in strict accordance with the French “Comité National de Réflexion Ethique sur l’Expérimentation Animale, n°15” and ethical guidelines for the care and use of the mice of the Plateau de Biologie Expérimentale de la Souris (PBES, UMS 3444) at Ecole Normale Supérieure (ENS, Lyon). The *in vivo* study was approved by the Committee on the Ethics of Animal Experiments of ENS de Lyon (approval number: ENS_2014_043 and ENS_2014_007).

### Isolation of human CD34^+^ cells from cord blood samples

Mononuclear cells were isolated from human cord blood by density centrifugation on Ficoll-Hypaque (Lymphoprep; Axis-shield) and CD34^+^ hematopoietic stem cells (HSC) were enriched using immunomagnetic beads according to the manufacturer instructions (CD34^+^ MicroBead Kit, Miltenyi Biotec, Bergisch-Gladbach, Germany). Purity (≥ 95%) and HLA-A2 expression were evaluated by FACS analysis using human PE-CD34 and HLA-A2 antibodies (Miltenyi Biotec). HLA-A2^+^ expressing cells were kept frozen until inoculation in newborn immunodeficient mice.

### Generation of humanized mice

NSG-HLA-A2/HDD(NOD.Cg-Prkdc^scid^ Il2rg^tm1Wjl^ Tg(HLA.A2.1)1Enge/SzJ) obtained from Jackson Laboratory were bred and maintained under pathogen-free conditions at the PBES. Newborn male and female NSG mice (2 to 5 days-old) were sub-lethally irradiated with 1.1 Gray (320 kV, 12.5 mA) from an X-ray irradiator (XRad-320, PXI Precision XRay) and intra-hepatically injected with 2 x 10^5^ human CD34^+^ HLA-A2^+^ HSC isolated from cord blood samples, in 30μl PBS [14]. Mice were daily monitored for signs of obvious suffering, such as weight loss, back arches and prostrated behavior. At week 6 post-engraftment, blood samples collected on ACD by retro-orbital puncture under Ketamine-Xylazine anesthesia were analyzed for the detection of human hematopoietic cells using hCD45, hCD3, hCD4, hCD8and hCD163 antibodies. At 10 weeks after engraftment, hu-mice in which more than 10% of huCD45^+^ cells were detected in peripheral blood, were subcutaneously engrafted with scaffolds containing or not allogeneic chondrocytes.

### Chondrocyte extraction and amplification

Human articular chondrocytes (HACs) were isolated from macroscopically healthy zones of osteoarthritic knee joints obtained from 9 donors undergoing total knee replacement. The study was performed in full accordance with local ethics guidelines, national and European Union legislation regarding human sample collection, manipulation and personal data protection (Ethics Committee for research with human samples, CODECOH: DC-2014-2325) and cartilage samples were collected after written informed consent of the donors. Chondrocytes were extracted as previously described [15]. Briefly, small slices of cartilage were digested in culture medium consisting of Dulbecco’s modified Eagle medium/Ham’s F12 (Gibco Invitrogen) with 0.06% bacterial collagenase A (Roche Applied Science) overnight. The cells were then seeded at a density of 1.5 x 10^4^ cells/cm^2^ on culture dishes with culture medium supplemented with 10% fetal calf serum (FCS) (Gibco), 100 mg/mL streptomycin and 100 U/mL penicillin (Invitrogen). Thirty-six hours after seeding, medium was refreshed and further supplemented with 5 ng/mL FGF-2 (R&D Systems) and 5 μg/mL insulin (Umuline Rapide, Lilly), namely the FI cocktail. The culture medium was replaced three times a week. At confluence, cells were trypsinized, counted with a hemocytometer and used for 3D culture.

### Cell culture in collagen sponges

The chondrocytes-collagen sponges (Symatese Biomatériaux, Chaponost, France) constructs were prepared as previously described [16]. Briefly, chondrocytes were seeded onto the sponges at the density of 13 x 10^6^ cells/cm^3^ and the sponges were incubated at 37°C for 2 hours. Culture medium containing 1% ITS (Insulin Transferrin Selenium; Gibco) and supplemented with 50 mg/mL 2-phospho-L-ascorbic acid (trisodium salt, Fluka) was then added in presence of 200 ng/mL of recombinant human BMP-2 (Dibotermine-alpha, drug form of BMP-2 contained in the kit InductOs, Wyeth), 5 μg/mL insulin (Umulin, Lilly) and 100 nM thyroxin T3 (Sigma). This cocktail was designated BIT. Medium was replaced every 2 days over a culture period of 3 weeks.

### Chondrocyte culture in agarose hydrogels

The chondrocyte-agarose constructs were prepared as previously described [17,18]. Briefly, trypsinized chondrocytes were embedded in 2% agarose (Seaplaque, Cambrex BioScience) at a density of 2 x 10^6^ cells/mL. Constructs were then placed in 6-well culture dishes and treated for 3 weeks with BIT culture medium. The culture medium was replaced every 2 days.

### Implantation of 3D constructs in hu-mice

Because a prerequisite of the study was to select a biomaterial which would generate minimal or no inflammatory response, acellular collagen sponges and agarose gels were first implanted in hu-mice for 4 weeks to evaluate their biocompatibility.

To evaluate more specifically the effect of allogeneic cells on the human immune response after transplantation/implantation of tissue-engineered cartilage, chondrocytes combined with collagen sponge or agarose hydrogel were implanted into subcutaneous pouches of hu-mice (1 construct/mouse) during 4 weeks. Chondrocytes from HLA-A2^+^ or HLA-A2^-^ donors were implanted in HLA-A2^+^ hu-mice according to the HLA combinations shown in Table 1.

**Table 1:** Combinations between scaffolds and human articular chondrocytes for implantation in HLA-A2^+^ hu-mice.

Number of mice (n)	HLA type of HACs	Scaffold
3	No cells	Collagen sponge
3	No cells	Agarose hydrogel
5	A2^+^	Agarose hydrogel
4	A2^-^	Agarose hydrogel
3	Not determined	Collagen sponge

After 4 weeks implantation, hu-mice were sacrificed and the implants were harvested and processed for immunohistochemistry analysis.

### Antibodies

For flow cytometry analysis, monoclonal antibodies provided by BD-Biosciences were used for cell staining in a 1% BSA 0.1% sodium azide PBS buffer: Pacific Blue-hCD45, FITC-CD3, PE-hCD8, PE-Cy7-hCD4, CD163-APC and HLA-A2-FITC. For western-blot and immunohistochemical analysis, Dr. J. Hartmann (Novotec) kindly provided polyclonal rabbit antibodies to human type II collagen (Ref 20211) [19]. Polyclonal rabbit antibodies to CD3 were from Dako (Ref A0452) and to actin were from Sigma (Ref A2066). Monoclonal antibody to CD68 was from Dako (Ref M0876). Secondary antibodies were alkaline phosphatase–conjugated anti-rabbit immunoglobulin (Ig)G (Bio-Rad) and horseradish peroxidase (HRP)-conjugated anti-rabbit or anti-mouse IgG (Cell Signaling and Vector ImmPRESS kit for Western blot and immunohistochemistry analysis, respectively).

### Western-blot analysis

After 3 weeks of *in vitro* culture followed or not by 4 weeks of implantation in mice, agarose hydrogels were harvested and processed for western-blot analysis as previously described [20]. Briefly, chondrocyte-agarose constructs were frozen in liquid nitrogen, freeze-dried and resuspended in Laemmli sample buffer. After boiling for 5 min in the presence of ß-mercaptoethanol, proteins were separated on 4–12% polyacrylamide gradient minigels (Biorad) and transferred to PVDF membranes (Millipore). The membranes were probed with the primary antibodies (dilutions: 1:3000 for type II collagen and 1:1500 for actin), washed and incubated with alkaline phosphatase-conjugated anti-rabbit IgG (dilution 1:5000). After multiple washes, bound antibodies were detected on X-ray films using a Bio-Rad Immun-star chemiluminescent substrate.

### Immunohistochemical analysis

Histologic examinations of the collagen sponges and agarose hydrogels seeded or not with chondrocytes were performed as previously described [16,17]. Briefly, the constructs were fixed for 24 h with formol acetic alcohol (AFA, Microm Microtech), dehydrated then embedded in paraffin. Hematoxylin and eosin counterstaining and immunohistochemical analysis was performed on 4- to 5-μm sections. Incubation with type II collagen (dilution 1:1000), CD3 (dilution 1:200) or CD68 (dilution 1:200) antibodies was followed by incubation with HRP-conjugated secondary antibodies. Sections were revealed with diaminobenzidine and observed using an ECLIPSE TI-E microscope (Nikon) coupled to a DS-Fi2 color camera. Acquisitions and treatment were performed with NIS-Elements imaging software (Nikon).

### Flow cytometry

At the time of their extraction from cartilage, 1 x 10^6^ chondrocytes were collected for HLA-A2 typing by flow cytometry analysis. The population of T lymphocytes and monocytes/macrophages was quantified 4 weeks after implantation of the scaffolds in mice. Peripheral blood cells were collected from the retroorbital venous sinus of hu-mice under isoflurane anesthesia. Mice were then sacrificed, spleens were collected and gently minced in PBS to obtain a single-cell suspension that was immediately frozen in FCS containing 10% DMSO and stored at -80°C. For flow cytometry analysis, cells were incubated for 30 min at 4°C in the dark in the presence of the relevant antibodies (Table 2). Cells were gated to exclude doublets. Compensations were realized using Miltenyi MACS Comp Beads. Fluorescence was acquired using FACSCanto II and BDSDiva software (Becton Dickinson Immunocytometry Systems, Mountain View, CA) and analyzed using FlowJo software (Treestar, Ashland, OR).

**Table 2:** Antibodies used for flow cytometry analysis.

Antigen	Conjugate	Isotype	Clone	Concentration	Origin	Company
hCD45	V450	IgG1	HI30	1/200	Mouse	BD Biosciences
hCD3	FITC	IgG2a	HIT3a	1/50	Mouse	BD Biosciences
hCD8	PE	IgG1	RPA-T8	1/50	Mouse	BD Biosciences
hCD4	PE-Cy7	IgG1	SK3	1/50	Mouse	BD Biosciences
hHLA-A2	FITC	IgG2b	BB7.2	1/25	Mouse	BD Biosciences
hCD34	PE	IgG1	581	1/50	Mouse	BD Biosciences
hCD19	APC	IgG1	HIB19	1/50	Mouse	BD Biosciences
hCD163	APC	IgG1	GHI/61.1	1/15	Mouse	Miltenyi Biotec
IgG1 isotype control	V450	IgG1	MOPC-21	1/50	Mouse	BD Biosciences
IgG2a isotype control	FITC	IgG2a	G155-178	1/50	Mouse	BD Biosciences
IgG1 isotype control	PE	IgG1	MOPC-21	1/50	Mouse	BD Biosciences
IgG1 isotype control	PE-Cy7	IgG1	MOPC-21	1/50	Mouse	BD Biosciences
IgG2b isotype control	FITC	IgG2b	27–35	1/50	Mouse	BD Biosciences
IgG1 isotype control	APC	IgG1	MOPC-21	1/50	Mouse	BD Biosciences
IgG1 isotype control	APC	IgG1	IS5-21F5	1/20	Mouse	Miltenyi Biotec

### Statistical analysis

For representation of the flow cytometry results, differences between experimental groups were analyzed using the Wilcoxon-Mann-Whitney U-test for nonparametric analysis. P < 0.05 was considered to be significant. The number of experiments performed is noted in the figure legends.

## Results

**Fig 1:**
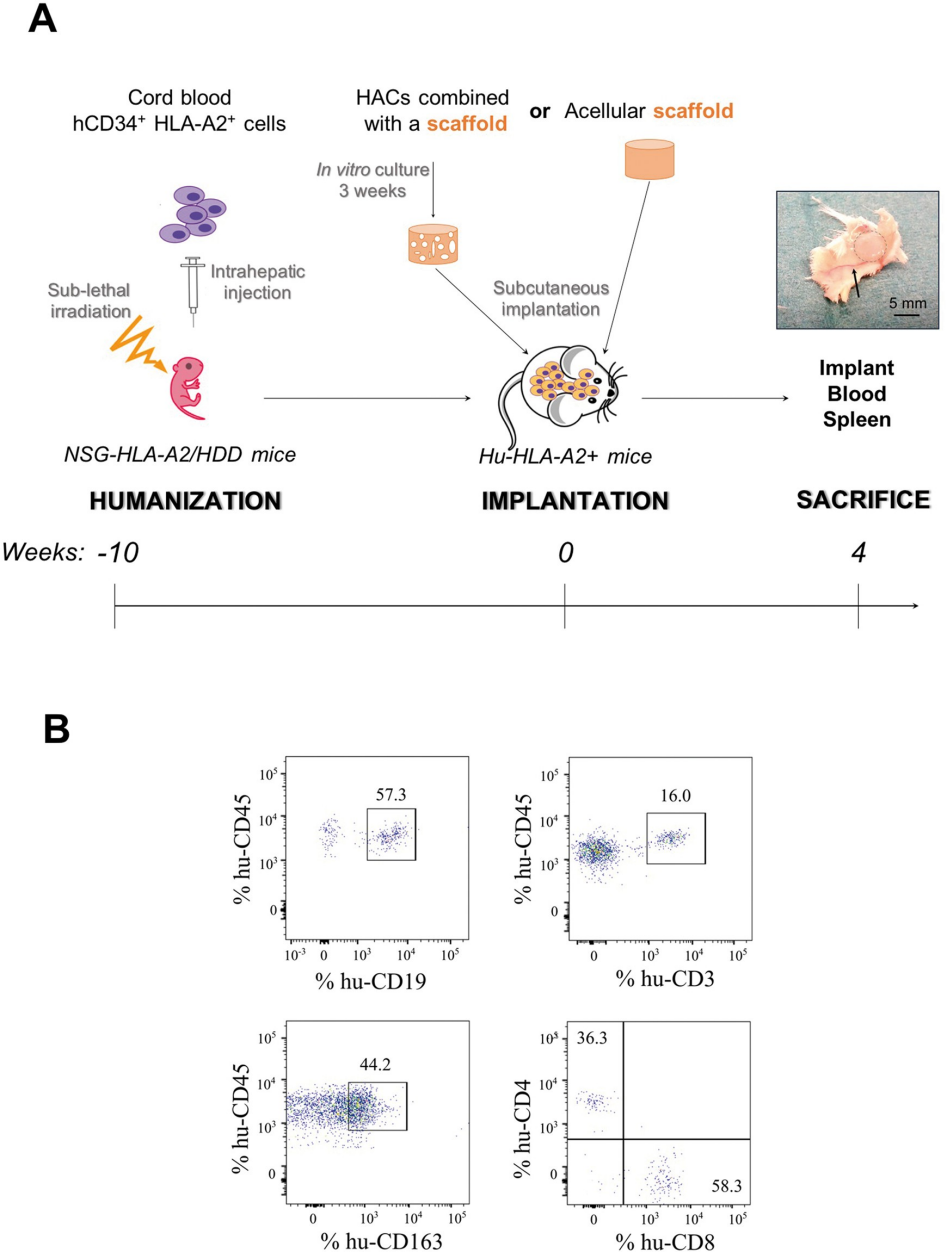
Experimental procedure of the study. (A) Newborn NSG mice were sub-lethally irradiated and intra-hepatically injected with human CD34^+^ hematopoietic stem cells (hCD34^+^ HSC) isolated from cord blood samples of HLA-A2^+^ donors. In parallel, human articular chondrocytes were extracted from knee joint cartilage of HLA-A2^+^ or HLA-A2^-^ donors, amplified and combined with scaffold. After 3 weeks of *in vitro* culture in the presence of BIT (200 ng/mL BMP-2, 5 μg/mL insulin, 100 nM T3), HLA-A2^+^ or HLA-A2^-^ cartilaginous constructs were subcutaneously implanted in the back of the HLA-A2^+^ humanized mice. 4 weeks after implantation, mice were sacrificed, cartilaginous constructs were harvested for western-blotting and immunohistochemistry analysis and spleens and blood samples were collected for flow cytometry analysis. The photograph shows macroscopic aspect of the implants just after harvesting from the mouse: the cartilaginous construct (black hatched circle) remains adherent to the skin and is vascularized (black arrow: blood capillary). (B) Human cell subpopulations in humanized mice. Flow cytometry analysis was performed 10 weeks after hCD34^+^ HSC inoculation. Cells were first gated to exclude the doublets. B (CD19^+^), T (CD3^+^) cells and macrophages (CD163^+^) were detected among human CD45^+^ cells. CD4 and CD8 cells were gated among T cells.

The overview of the experimental procedure pursued during our study is described in Fig 1A. The first step consisted in intra-hepatically inoculation of HLA-A2^+^ CD34^+^ HSC in irradiated newborn NSG-HLA-A2/HHD mice. Ten weeks after HSC inoculation, the analysis of human cells in mice indicated that immune reconstitution was achieved (10–40% of human CD45^+^ cells) with known frequencies of B (CD19^+^) and T (CD3^+^) cells and macrophages (CD163^+^) (Fig 1B). At the time of the sacrifice, human CD45^+^ cells as well as major subsets of human immune cells were also detected in the thymus, mesenteric lymph nodes, and bone marrow of these hu-mice (data not shown), as previously described in studies using this hu-mice model [21,22]. This reconstitution of immune cell populations demonstrated that a human-like immune system was achieved, which validated hu-mouse as a model to assess biocompatibility of human allogeneic cartilage constructs.

### Response of hu-mice to acellular scaffolds

**Fig 2:**
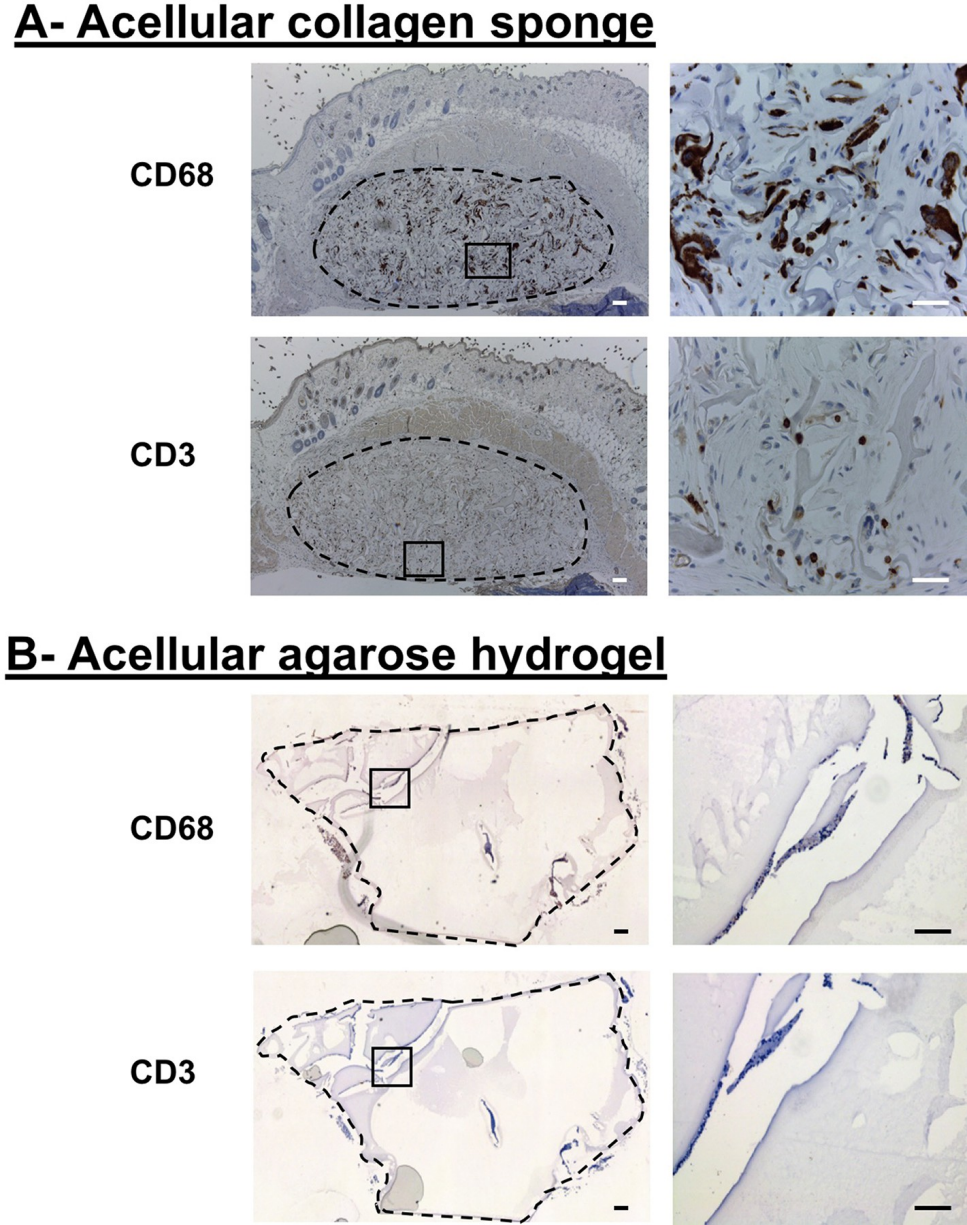
Infiltration of immune cells in acellular collagen sponge. Immunostaining of human CD3^+^ T cells and CD68^+^ macrophages in (A) acellular collagen sponge and (B) in acellular agarose gels, 4 weeks after implantation in hu-mice. Right panels show high magnifications of the zones framed by solid lines in the left pictures. Scale bars are 100 μm.

Because our main objective was to evaluate the immune response to allogeneic chondrocytes-seeded scaffolds and not to the biomaterial itself, pilot studies were first realized with acellular scaffolds to select a support giving minimal immune reaction: we compared two types of scaffolds routinely used in our laboratory, collagen sponges [16] and agarose hydrogels [20]. These scaffolds were separately subcutaneously grafted in the back of hu-mice for 4 weeks then harvested and processed for immunohistochemistry analysis. The implants were surrounded by blood capillaries indicating their potential vascularization and contact with the peripheral immune system. Of note, the close vicinity of blood capillaries was particularly more easily recorded by photography in the case of the collagen sponges since they remained intimately attached to the skin after dissection (Fig 1A), unlike the agarose gels. At the microscopic level, massive cell infiltration inside in the collagen sponge was observed (Fig 2A). Immunohistochemistry analysis indicated the presence of human hematopoietic cells: CD3^+^ lymphocytes and CD68^+^ macrophages (Fig 2A), a sign of slight local inflammatory response. In contrast, cell infiltration or staining for T lymphocytes and macrophages was not detected in acellular agarose hydrogels (Fig 2B). Analysis of peripheral tissues revealed that proportions of T-cells were similar to those observed in non-implanted hu-mice (peripheral blood: 9–30%; spleen: 4–6%, data not shown) confirming the absence of local and peripheral inflammation provoked by the agarose scaffold.

These results led us to deepen our evaluation of the biocompatibility of allogeneic tissue-engineered cartilage in humanized mice by using agarose as scaffold.

### Response of hu-mice to allogenic HACs combined with agarose hydrogel

**Fig 3:**
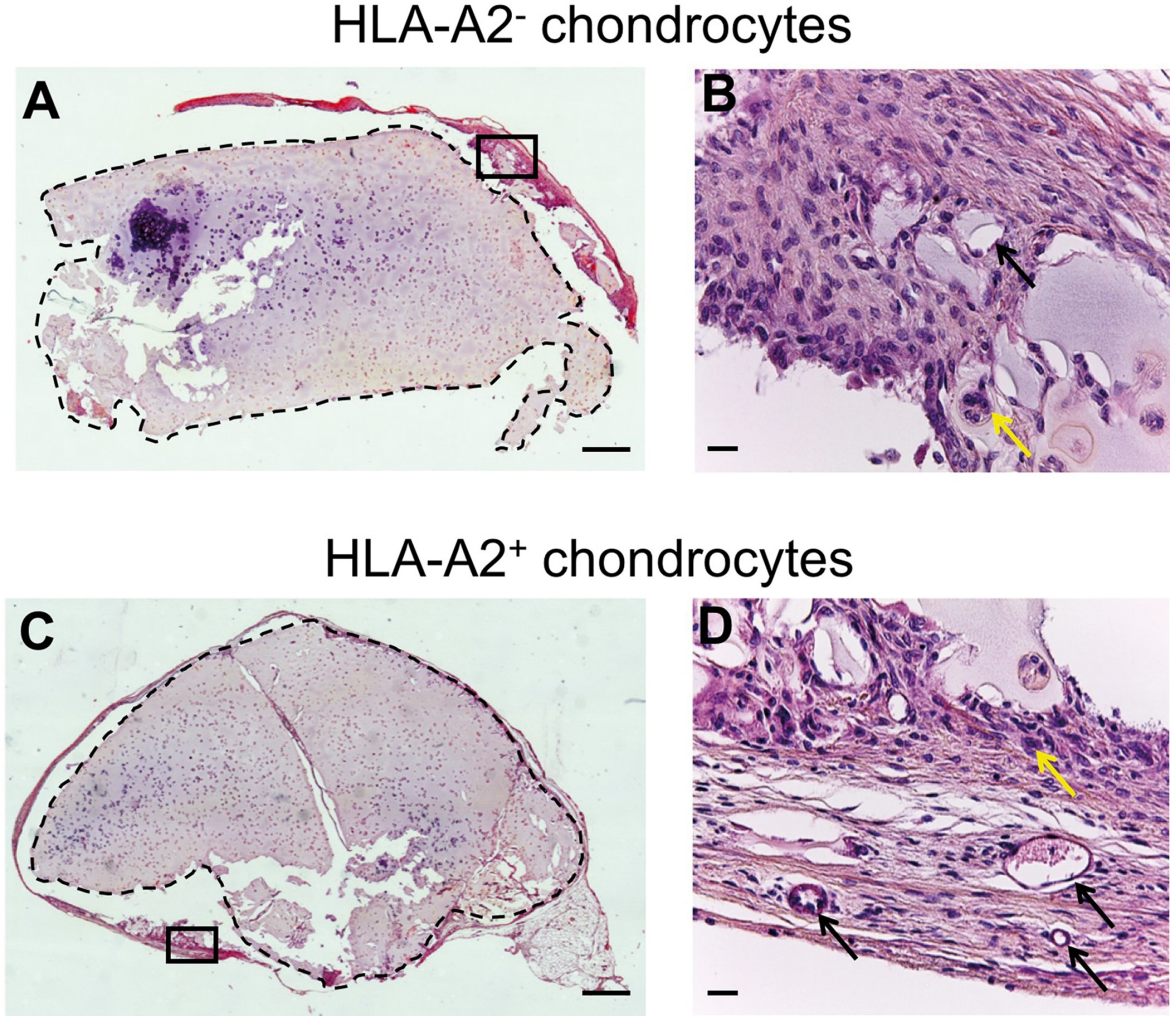
Stability of HLA-A2^-^ and HLA-A2^+^ chondrocyte-agarose constructs implanted in HLA-A2^+^ hu-mice. Haematoxylin and Eosin (H&S) staining of HLA-A2^-^ (A, B) and HLA-A2^+^ (C, D) human articular chondrocytes embedded in agarose hydrogels. These constructs were cultured in presence of BIT for 3 weeks *in vitro* then implanted in HLA-A2^-^ hu-mice for 4 weeks. (B) and (D) show high magnifications of the zones framed by solid lines at the periphery of the implants in (A) and (C), respectively. These zones are fibrous tissues rich in fibroblasts. Black arrows indicate the presence of micro-vessels and yellow arrows the presence of giant cells composed of fused macrophages. (A, C) scale bars are 500 μm and (B, D) scale bars are 20 μm.

**Fig 4:**
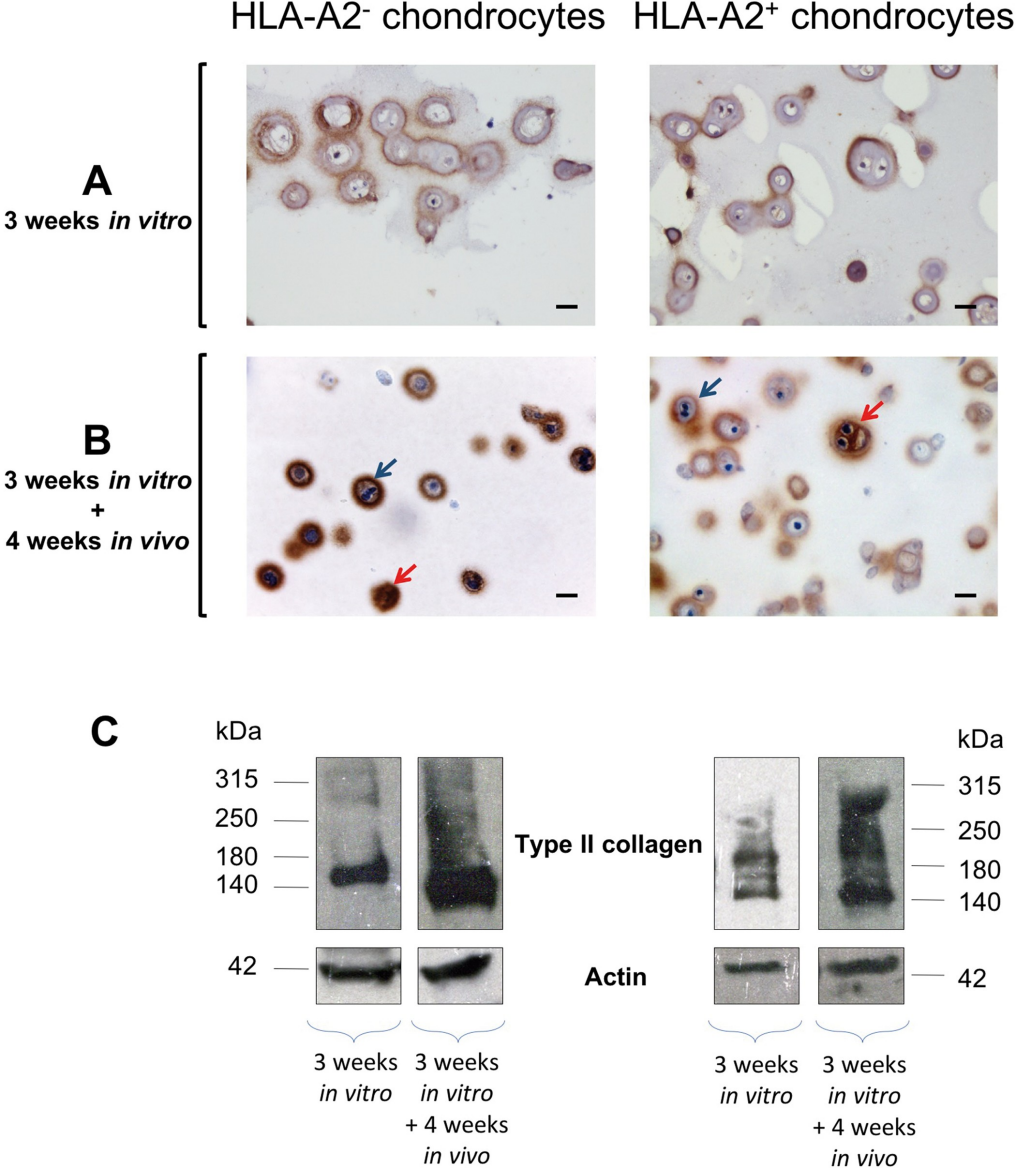
Cartilage-characteristic matrix is produced *in vitro* in HLA-A2^-^ and HLA-A2^+^ chondrocyte-agarose constructs then accumulates with time in HLA-A2^+^ hu-mice. (A, B) Type II collagen immunostaining of HLA-A2^-^ and HLA-A2^+^ chondrocyte-agarose constructs after 3 weeks of *in vitro* culture (before implantation) and after 3 weeks of *in vitro* culture followed by 4 weeks of *in vivo* implantation (at the end of the implantation period) in HLA-A2^-^ hu-mice. Note that cells exhibit more intense intra- and peri-cellular staining after, rather than before, implantation. (C) Western blot analysis of type II collagen synthesis before and 4 weeks after implantation. Note that type II collagen was more abundant in the cartilage disks after the implantation period, in accord with the immunohistochemical data. This abundant production also explain why bands are smeared. Scale bars are 20 μm.

Because the main risk of graft rejection is non-compatibility between the HLA systems of the donor and the recipient, we tested the importance of HLA matching between HACs and hu-mice. Briefly, human chondrocytes (isolated from HLA-A2^-^ or HLA-A2^+^ donors) were amplified and embedded in agarose hydrogel using the combination of the FI and BIT cocktails that was previously shown to be effective for cartilage matrix production of HACs in collagen sponges [16,23]. These HLA-A2^-^ or HLA-A2^+^ constructs were implanted in HLA-A2^+^ hu-mice during 4 weeks, then mice were sacrificed and implants were harvested and processed for immunohistochemistry and Western-blotting analysis. In fact, we obtained equivalent results with the HLA-A2^-^ and HLA-A2^+^ constructs. First, hematoxylin and eosin (H&E) staining of the constructs showed good maintenance of structural integrity, with very thin surrounding fibrous capsule formation (Fig 3A and 3C). Round to oval shaped structures characteristic of micro-vessels and multinucleated cells typical of macrophages were observed at the periphery of the implants (Fig 3B and 3D). In hydrogels, chondrocytes displayed a typical round morphology and immunostaining revealed at the cell periphery an accumulation of type II collagen, the most abundant protein found in native cartilage (Fig 4). These observations confirmed that cells expressed a differentiated chondrocyte phenotype. Interestingly, we observed that type II collagen staining at the cell periphery was denser in the constructs that were implanted, in comparison with the constructs that were cultured *in vitro* only (Fig 4A and 4B). To gain a more quantitative appreciation of type II collagen production, chondrocyte-agarose constructs were analyzed by Western-blotting and our results clearly indicated that type II collagen accumulated with time in hydrogel, during the implantation phase in mouse (Fig 4C).

**Fig 5:**
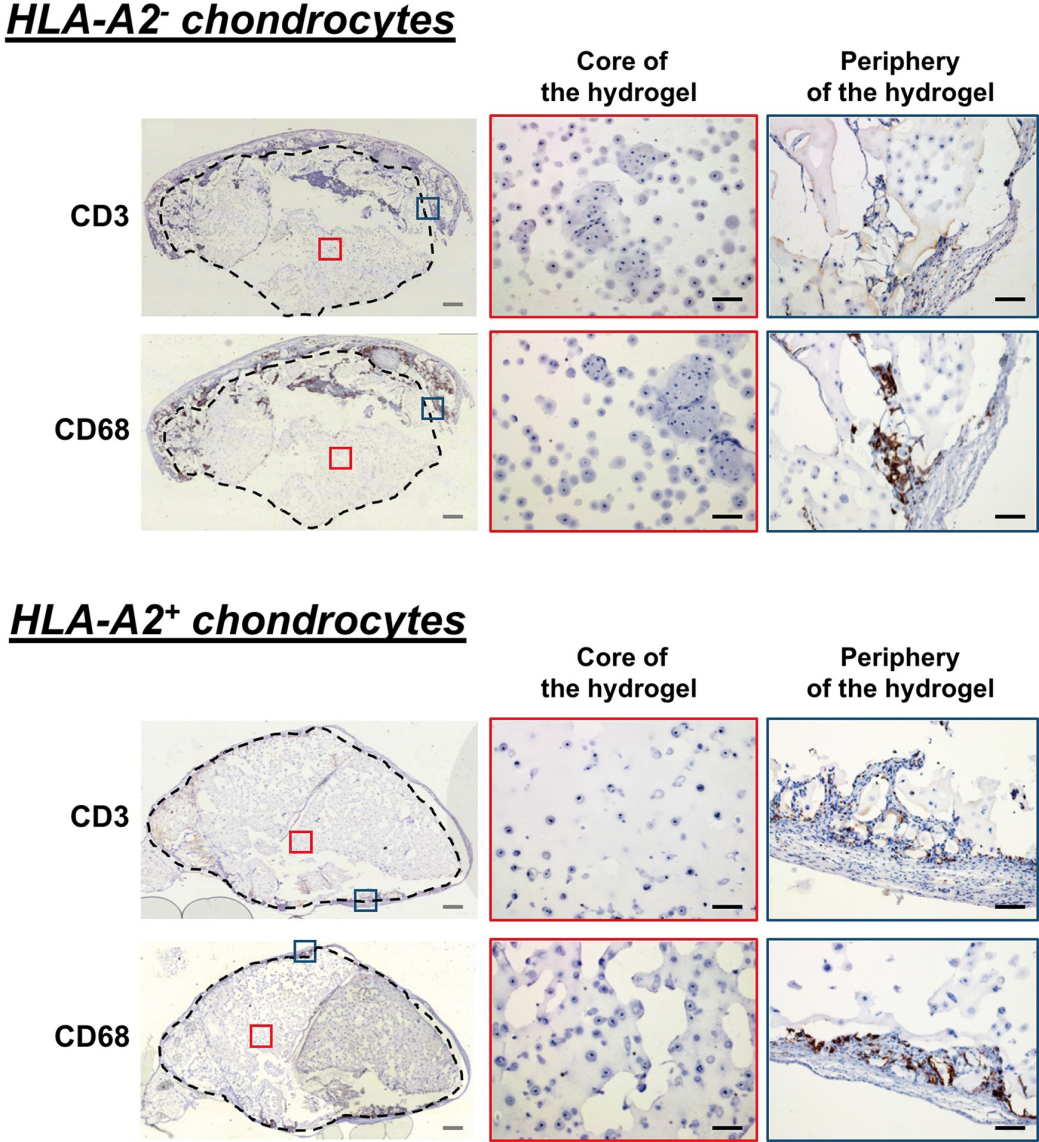
Immune cells do not infiltrate agarose gels containing HLA-A2^-^ or HLA-A2^+^ chondrocytes. Immunostaining of human CD3^+^ T cells and CD68^+^ macrophages in the core (red squares) and at the periphery (blue squares) of HLA-A2^-^ or HLA-A2^+^ chondrocyte-agarose constructs that were implanted for 4 weeks in HLA-A2^+^ hu-mice. Note sparse staining of CD3^+^ and CD68^+^ cells in the fibrous membranes surrounding the implants. Black scale bars are 500 μm. White scale bars are 100 μm.

No sign of inflammatory response was detected in the core of the chondrocyte-agarose constructs, regardless of the HLA-A2 type of chondrocytes. However, our immunohistochemistry analysis showed sparse staining of CD3^+^ and CD68^+^ cells in the fine fibrous membranes surrounding the implants (Fig 5). Since this close vicinity of CD3^+^ and CD68^+^ cells with the chondrocytes could favor an inflammatory and/or immune response, we then closely examined the presence of T lymphocytes and macrophages in the peripheral blood or spleen of the hu-mice that were implanted with the agarose constructs.

### Peripheral response of hu-mice implanted with allogeneic HACs seeded in agarose hydrogels

**Fig 6:**
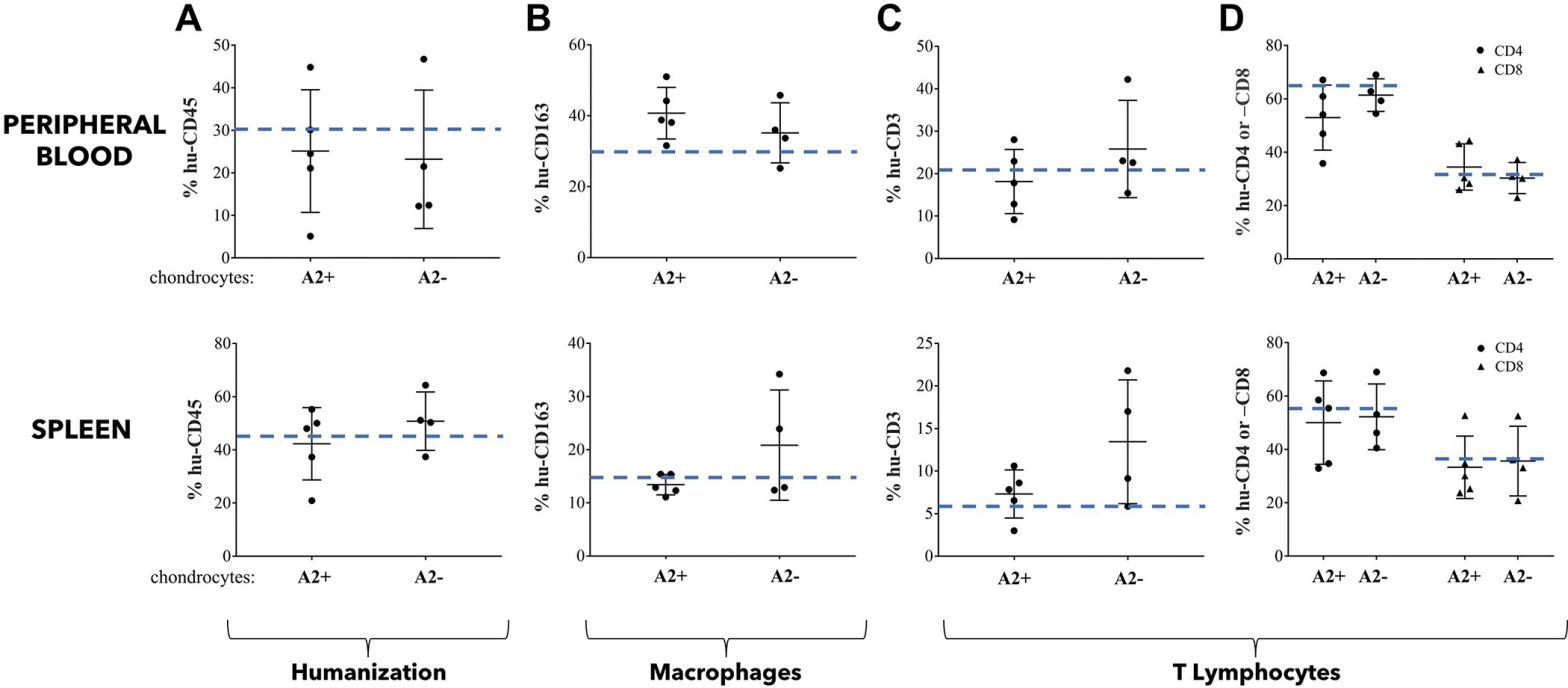
Allogeneic cartilage constructed in agarose gel do not trigger peripheral inflammatory/immune response. Flow cytometry analysis of characteristic human cell surface markers in peripheral blood and spleen of HLA-A2^+^ mice that were implanted for 4 weeks with HLA-A2^+^ (A2+) or HLA-A2^-^ (A2-) chondrocyte-agarose constructs. (A) Percentage of cells expressing CD45. (B) Percentage of cells expressing CD3 (total T lymphocyte population) among CD45 positive cells. (C) Comparison of subpopulations of T lymphocytes, CD4^+^ and CD8^+^. (D) Determination of the proportion of cells expressing the CD163 marker (monocytes / macrophages) among human cells (hCD45 positive cells). Data are presented as mean±SD. For each graph, the dashed blue line represents the average percentage of cells expressing the cell surface marker when hu-mice were implanted with acellular agarose hydrogels.

First of all, we monitored by flow cytometry analysis the level of humanization of mice used in our different experiments. We found about 30% and 45% of hCD45^+^ cells in peripheral blood and spleen, respectively, in mice implanted with chondrocyte-agarose constructs regardless of HLA-A2 type of chondrocytes. Of note, these percentages were in the same range as those observed in mice implanted with acellular agarose hydrogels (Fig 6A, dotted blue line).

We then analyzed the impact of allogeneic HACs-seeded agarose constructs on the distribution of the lymphoid cell subpopulations in peripheral blood and spleen. Globally, our flow cytometry analysis showed similar profiles of expression of hCD3^+^, hCD4^+^, hCD8^+^ T lymphocytes and hCD163^+^ macrophages, in peripheral blood or spleen of hu-mice implanted with cartilage disks containing HLA-A2^+^ or HLA-A2^-^ chondrocytes (Fig 6B–6D). These expression profiles were not significantly different from those obtained after implantation of acellular hydrogels (dotted blue lines, Fig 6).

Taken together, our results indicated that implantation of allogeneic chondrocytes seeded in agarose hydrogel did not stimulate peripheral inflammation in hu-mice, regardless of the HLA-A2 type of chondrocytes.

## Discussion

The main goal of the present study was to assess the biocompatibility and stability of allogeneic human cartilage constructs by using an *in vivo* model. For this, we relied on hu-NSG-HLA-A2 mice displaying a human hemato-lymphoid system [24], in order to examine the reaction of human immune cells to implantation of scaffold-based human cartilage constructs.

Firstly, our analysis of the responsiveness of hu-mice to acellular scaffolds (porous collagen sponge and non-porous agarose hydrogel) showed infiltration of human CD3^+^ T lymphocytes and CD68^+^ macrophages in sponge but not in gel. This finding is consistent with previously reported observations of mild and transitory host tissue reaction generally observed with collagen sponges [25–27]. The high porosity of collagen sponges most likely explains the cellular invasion, leading to an early and weak inflammatory reaction that will not evolve over time, as reported by Anselme *et al*. [25]. In contrast, agarose can be considered as a non-porous material at the cell level. Indeed, the pore size in the collagen sponges used in this study is about 100 μm [23] while the pore size in 2% agarose gel has been previously estimated by different methods to be around 200 nm [28,29]. Thus, the average diameters of human macrophages and T lymphocytes of about 20 μm and 7 μm, respectively [30,31] should not allow these cells to migrate across the hydrogel. In agreement with this, we did not observe infiltration of cells in agarose hydrogel. However, previous reports indicated that this type of scaffold material can trigger a slight foreign body response with the presence of macrophages in areas surrounding the implant with the formation of a thin fibrous capsule around the hydrogel [32,33], as observed in our study. Since cellular immunity is activated by T lymphocytes and macrophages that secrete soluble and diffusible factors such as immunoglobulins or complement, there is still the possibility that chondrocytes are exposed to humoral immunity. A previous *in vitro* study reported that chondrocytes can be damaged in presence of high complement concentration [34], but our results indicate that it does not seem to be the case in agarose.

On top of that, it should be added that cartilage matrix deposited by the cells in the pericellular space likely plays a role in masking cell surface molecules. In the same line, Freed *et al*. [35] found that chondrocyte-PGA allografts do not induce immune response in rabbit and hypothesized that cell surface antigens are sequestered in cartilage matrix produced during *in vitro* culture. Our data are also in agreement with other transplantation studies using allogeneic chondrocytes embedded in different hydrogels, where no sign of rejection or cell invasion was detected with respect of the cartilage that was reconstructed [4,36,37]. Therefore, the combined contribution of agarose and cartilage matrix most likely explains why we did not detect activation of the peripheral immune system although inflammatory/immune cells were present in close proximity of the chondrocytes seen at the periphery of the agarose implants (S1 Fig). This highlights the fact that, beyond the choice of the scaffold itself, the status of the phenotype of the chondrocytes embedded in the scaffold is certainly of equal importance to ensure biocompatibility of allogeneic cartilage constructs. Here, our study shows that HACs were able to build an extracellular matrix rich in type II collagen in agarose. It should be specified that the chondrocytes were certainly partially dedifferentiated at the time they were mixed with agarose, after amplification on plastic. It is well-known that amplification of chondrocytes induces their dedifferentiation [38] and we have previously reported that the FI cocktail, besides its proliferative effect on HACs, also stimulates their dedifferentiation [16]. We have also shown that, after FI-induced dedifferentiation, the BIT cocktail can drive redifferentiation of human nasal chondrocytes seeded in self-assembling peptide hydrogel [39] and of HACs seeded in porous collagen sponge [16,23], with production of type II collagen and other cartilage-characteristic molecules such as type IX collagen and sulfated proteoglycans.

Then, in order to better evaluate contribution of the newly synthesized extracellular matrix in ensuring biocompatibility of allogenic tissue-engineered cartilage, we exploited the humanized mouse model to implant allogenic BIT-treated HACs seeded in porous scaffold. Hu-mice were implanted with collagen sponge based-cartilage constructs generated by using the same protocol as described in Fig 1. Four weeks after implantation, we observed massive cell infiltration but the implants retained their structural integrity (S2A, S2B and S2C Fig) and chondrocytes displayed a differentiated phenotype with accumulation of type II collagen around them (S2D, S2E and S2F Fig). Macrophages and T lymphocytes were detected in the constructs (S2G, S2H, S2I and S2J Fig) but of note, the densities of staining corresponding to CD3 and CD68 positive cells appeared equivalent to those detected in acellular collagen sponges (Fig 2A). These observations revealed that addition of well-differentiated allogeneic chondrocytes does not exacerbate the mild host tissue reaction provoked by porous sponge, most likely because the newly-synthesized pericellular matrix isolates them from immune reaction. These results highlight the barrier role of the extracellular matrix (ECM). Studies have shown that scaffolds based on ECM components of the native cartilage, such as cartilage-specific glycosaminoglycans [40,41], promote the metabolic activity of chondrocytes and production of ECM, which in turn could improve the protection of allogeneic chondrocytes against an immune response.

Several studies on cartilage repair have previously highlighted the possibility of using allogeneic cartilage constructs but these investigations were restricted to animal models such as rabbits and rats [37,42,43]. Here, using hu-mice, we found that the presence of allogeneic chondrocytes seeded in hydrogel or in collagen sponge do not stimulate the hemato-lymphoid system. The only host response registered was local and caused by collagen sponge. Although it should be kept in mind that the immune system developed in hu-mice may partially represent true human immune system, our data support that allogeneic chondrocytes represent valuable candidate cells for tissue engineering of cartilage.

## Conclusions

We have utilized the humanized mouse (hu-mice) model, naturally derived biomaterials and allogeneic human chondrocytes to investigate the possibility of using allogeneic cartilage constructs for human cartilage repair. This work reveals that allogeneic chondrocytes represent indeed suitable alternative cells to autologous chondrocytes and paves the way to an off-the shelf procedure for cartilage tissue engineering. Such an approach should more easily satisfy the growing clinical demand since cartilage tissue banking should provide easy access to chondrocyte reservoirs needed to construct cartilage. Since tissue sampling from patient would not be required, allogeneic grafting would also simplify the original surgical procedure using autologous chondrocytes [44,45]. In this context, scaffold biocompatibility is obviously required. Here, we have shown in particular that agarose hydrogel behaves as an excellent stealth-like material for allogeneic chondrocytes. Interestingly, agarose has already been used to create cartilage patches in clinical trials [46] but other types of hydrogels could be considered for cartilage engineering such as fibrin [47,48], PEG/hyaluronic acid-based hydrogels [49], silk [50] or cellulose-based hydrogels [51,52] proposed as medical devices. Moreover, other hydrogels are proposed for their potential as medical device not only for tissue repair but also for the regulation of inflammation. Innovative formulations based on hyaluronic acid (HA) promote tissue regeneration by stimulating resident stem cells [53,54]. D'Agostino *et al*. [55] have also demonstrated that, by creating a high-low molecular weight HA complex, healing is improved while limiting the inflammation normally induced by HA alone. In the same vein, Stellavato *et al*. [41] developed a new chondroitin sulfate-based hydrogel which preserves the chondrocyte phenotype and presents biological activity in respect to inflammation by reducing inflammatory response induced by interleukin-1ß. Thus, such hydrogels represent also potential candidate scaffolds for cartilage repair. The selection of soluble factors resulting in efficient chondrocyte differentiation and synthesis of extracellular matrix should also greatly contribute to make cell surface antigens undetectable by the host immune system, at the time of implantation. This is supported by our results using allogeneic chondrocytes and collagen sponges. This latter point is interesting since collagen sponges are also envisaged by several groups for cartilage tissue-engineering [56,57,58]. Finally, we demonstrated for the first time that hu-mice represent a useful preclinical tool to investigate the response of human hemato-lymphoid cells to engineered cartilage constructs.

## Supporting information

S1 Fig Presence of CD68^+^ macrophages in close proximity of the chondrocytes at the periphery of the agarose implants. Scale bar is 10 μm.
(TIF)

S2 Fig Immunogenicity of allogeneic cartilage constructed in collagen sponge. Collagen sponges seeded with human articular chondrocytes were cultured for 3 weeks *in vitro* in the presence of BIT cocktail then implanted in humanized mice for 4 weeks. (A-B-C) Haematoxylin and Eosin (H&S) staining of implants before and after implantation. Note the presence of a micro-vessel (black arrow) at the periphery of the implant and the increase in cell density with infiltration of macrophages (white arrows) in the core of the sponge after implantation. (D-E-F) Immunostaining of human type II collagen showing intracellular synthesis (white arrows) and extracellular matrix deposition. (G-H) Immunostaining of human CD68^+^ T cells. (I-J) Immunostaining of human CD3^+^ macrophages. (G, I) scale bars are 200 μm; (A, B, D, E) scale bars are 100 μm; (C) scale bar is 25 μm and (F, H, J) scale bars are 10 μm.
(TIF)
