# Supplementary figures and images for: Evaluation of the biocompatibility and stability of allogeneic tissue-engineered cartilage in humanized mice
Source: PLoS One. 2019 May 20;14(5):e0217183. doi: 10.1371/journal.pone.0217183 (PMC6527235; doi:10.1371/journal.pone.0217183)

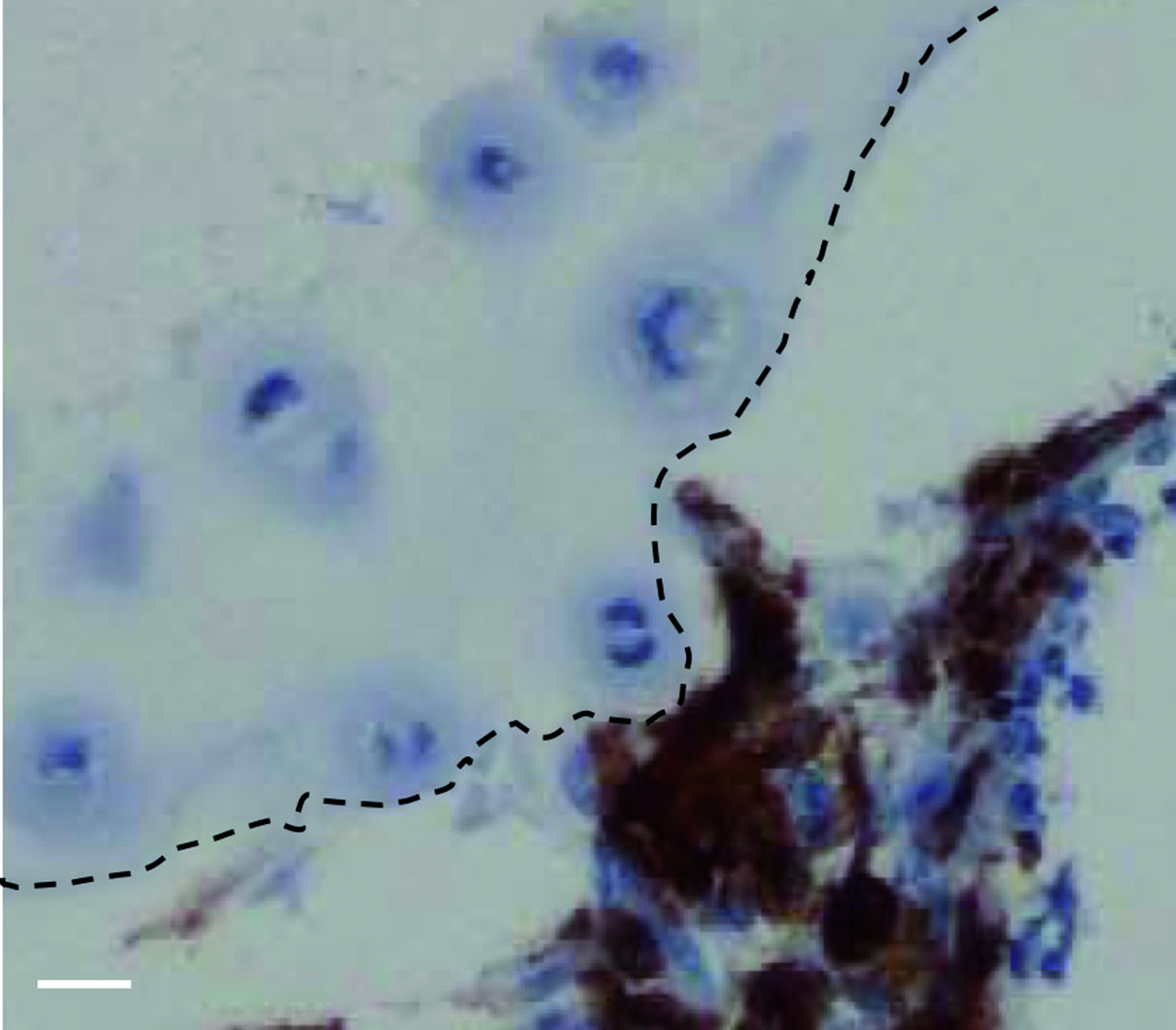

Supplement: S1 Fig — Scale bar is 10 μm. (TIF) [file pone.0217183.s001.tif]

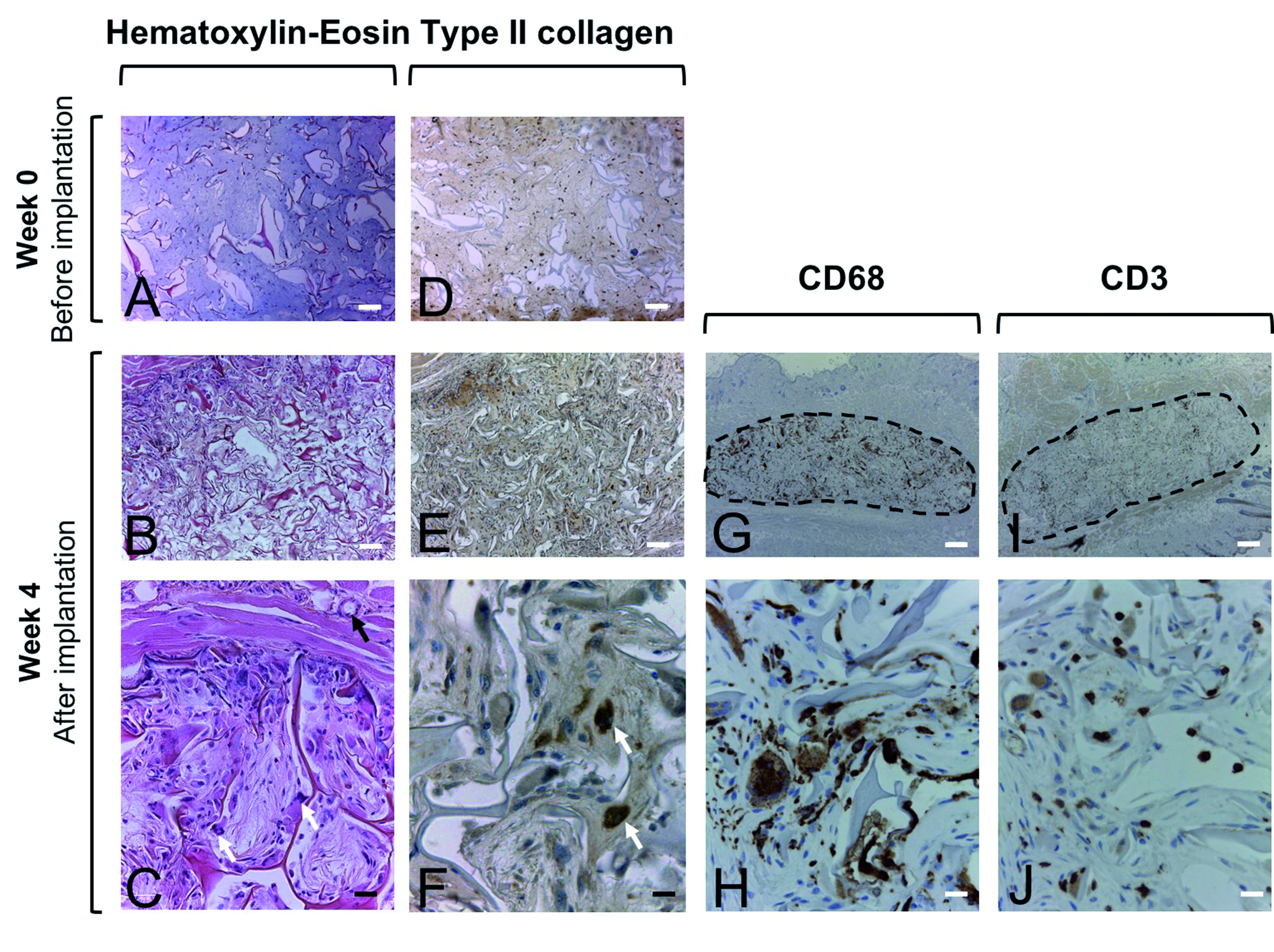

Supplement: S2 Fig — Collagen sponges seeded with human articular chondrocytes were cultured for 3 weeks in vitro in the presence of BIT cocktail then implanted in humanized mice for 4 weeks. (A-B-C) Haematoxylin and Eosin (H&S) staining of implants before and after implantation. Note the presence of a micro-vessel (black arrow) at the periphery of the implant and the increase in cell density with infiltration of macrophages (white arrows) in the core of the sponge after implantation. (D-E-F) Immunostaining of human type II collagen showing intracellular synthesis (white arrows) and extracellular matrix deposition. (G-H) Immunostaining of human CD68+ T cells. (I-J) Immunostaining of human CD3+ macrophages. (G, I) scale bars are 200 μm; (A, B, D, E) scale bars are 100 μm; (C) scale bar is 25 μm and (F, H, J) scale bars are 10 μm. (TIF) [file pone.0217183.s002.tif]
